# 

**Published:** 1879-07

**Authors:** 


CONTENTS.

**CONTRIBUTIONS.** PAGE.

Heels: Et Id Omne Genus, BEN. H. PRATT.........143
As well as we know, J. J. KEYES.........146
Do the Fittest really Survive?, AUSBURN TOWNER......148
Come! What say you?, THOS K. BEECHER............151

**MEDICAL ITEMS AND NEWS.**

Vomiting During Pregnancy—Chloral Liniment—Chloral Ointment—Ointment for Piles—Cough Mixture—Lotion for Sore Nipples—Salicylic Acid against Tænia—Chloroform Narcosis—Eucalyptus for Cold in Head—Sedative action of Quinia on Neck of Bladder—Hypodermic Injection of Salicylic Acid in Erysipelas—Phosphorus in Sciatica—Chloroform—Rheubarb in Preventing Nausia from Morphia—New Mode of using Ether—Nitrate of Pilocarpine in Pleuro Pneumonia—Zinc Oxide in Diorrhœa—Cactus and Heart's Ease—Aromatic Liquid Pepsin—Chloral Suppositories—Chronic Bronchitis—Compound Bismuth Powder—Bromide of Ethyl in Neuralgai and Sleeplessness—Hemp Smoking in Tetanus—Benzoate of Lithia—German Treatment of Croup and Diphtheria—Capsicum and Quinine—Damiana—Caffeine as a Diuretic and Stimulant—Eczema of the Head—Diphtheria and Chlorine Water—Milk Diet in Inflammation of Bladder—Surgical Treatment of Anasarca—Enementa of Chloral in Sick Headache—Covering Taste of Cod Liver Oil—Spirit of Walnut for Vomiting—Detection of Glucose in Urine—New Blistering Plaster—Mercury for Hypodermic Use—For External Use—Croup Cured by Hypodermic Injection of Sulphate of Atropia—Salicylate of Soda in Acute Rheumatism.................153 to 161

**OUR CORRESPONDENCE.**

Eight Letters with Replies.................162 to 163

**HOUSEHOLD HINTS AND RECIPES.**

Waffles—Color Red the Edges of Books—Liquid Glue Sawdust in Art—Moth Destroyer—Corn Bread—Moths in Furniture—Dangers of Mouldy Bread—Test for Water—Keep Beds free of Bugs........164 to 165

**EDITORIAL.**

THE PHYSICIAN.........166

CATCH THE MOTH.........167

DON'T.........168

CAPITAL PUNISHMENT.........168

**EDITORIAL CHIT-CHAT.**

"Bodines"—Delinquents—Camping Book—Bushels of Cats—Cremation Society—Beef Wine and Iron—The Loan Exhibition—Lawn Mower—Club 101—Unconscious Hilarity—Home Prescriptions—Dixey on Art—Politeness—Dwight Inquest—Be Cautious—Sunday Newspapers—Warner's Pills and Parvules..169 to 170

